# Cognitive complaints compared to performance on a mental state screening test in elderly outpatients

**DOI:** 10.1590/S1980-57642009DN20100010

**Published:** 2008

**Authors:** Renata Kochhann, Ana Luiza Camozzato, Cláudia Godinho, Maria Otília Cerveiro, Letícia M.K. Forster, Márcia Lorena Fagundes Chaves

**Affiliations:** 1 Dementia Clinic, Neurology Service, Hospital de Clínicas de Porto Alegre.; 2 Medical Sciences Post-Graduation Course, UFRGS School of Medicine.; 3 Internal Medicine Department, UFRGS School of Medicine.

**Keywords:** cognitive complaint, aging, elderly, Mini Mental State Exam, cognitive decline associated with aging, dementia, depression, memory, queixa cognitiva, envelhecimento, idoso, Mini Exame do Estado Mental, declinio cognitivo associado ao envelhecimento, demencia, depressão, memória

## Abstract

Memory and other cognitive complaints are common in the elderly population. However, the clinical meaning of these complaints remains controversial.

**Objectives:**

The goal of this study was to investigate the association between cognitive complaints and performance on a mental state screening test in elderly patients attended for the first time at the Neurogeriatric and Dementia (NGA) Outpatient Clinic within a major University hospital.

**Methods:**

Two hundred patients referred to the NGA Clinic during 2005, 2006 and 2007 first semesters participated in the study. The variables of interest were:

(a) source of and reason for referral;
(b) whether or not they had previously been evaluated with the screening test (Mini Mental State Exam - MMSE) by their physicians before referral to our specialized clinic;
(c) cognitive complaints; and
(d) performance on the screening test (MMSE) at the NGA Clinic.

**Results:**

The main reason for referral to the NGA clinic was cognitive complaints 63% (N=126), where only 5% (N=10) of the referred patients had been previously evaluated by the cognitive screening test (MMSE or equivalent). Of the 135 patients who presented cognitive complaints during the first appointment, 52 (38%) presented MMSE scores below the education-adjusted cut-off. No association between cognitive complaint and performance on the MMSE during the first evaluation at the NGA Clinic was observed (χ^2^=3.04, p=0.1).

**Conclusions:**

Although cognitive complaints among elders should not be disregarded, the mental state screening evaluation is crucial for the detection of clinically significant cognitive impairment.

## Abstract (pt)

Memória e outras queixas cognitivas são comuns na população idosa. No entanto, o significado clínico destas queixas permanece controverso.

**Objetivo:**

O objetivo deste estudo foi investigar a associação entre queixa cognitiva e desempenho em um teste de rastreio do estado mental em pacientes idosos que consultavam pela primeira vez no Ambulatório de Neurogeriatria e Demência (NGA) de um grande hospital universitário.

**Métodos:**

Duzentos pacientes que foram encaminhados ao Ambulatório de Neurogeriatria durante os primeiros semestres de 2005, 2006 e 2007 participaram do estudo. As variáveis de interesse foram:

(a) fonte e razão do encaminhamento;
(b) existência de avaliação, com um teste de rastreio (Mini Exame do Estado Mental - MEEM), pelos seus médicos assistentes prévia ao encaminhamento ao NGA;
(c) queixa cognitiva na consulta do NGA, e
(d) desempenho no teste de rastreio (MEEM) no Ambulatório NGA.

**Resultados:**

O principal motivo de encaminhamento ao ambulatório de NGA foi queixa cognitiva 63% (N=126), somente 5% (N=10) dos pacientes encaminhados foram submetidos a algum teste de rastreio cognitivo prévio (MEEM ou equivalente). Dos 135 pacientes com queixa cognitiva durante a primeira avaliação, 52 (38%) apresentavam escores do MEEM abaixo do ponto de corte, ajustados para escolaridade. Nenhuma associação entre queixa cognitiva e desempenho no MEEM durante a primeira avaliação no ambulatório de NGA foi observada (χ^2^=3.04, p=0.1).

**Conclusões:**

Embora, queixas cognitivas entre idosos não devem ser ignoradas, avaliação, através de rastreio do estado mental, é crucial para detecção de comprometimento cognitivo clinicamente significativo.

Memory and other cognitive complaints are common in 60-year-olds and over, especially upon comparing their current and past performance.^1^ The range of prevalence of memory complaints is large and estimated to be around 22 to 56% in the geriatric population.^2^ In a cohort study with 524 elderly individuals, the prevalence rate observed was 27%.^3^ Another study involving a larger sample (n=2537) of elderly without dementia or depression reported a prevalence rate of 34%.^4^ The frequency of memory complaints increases with age, occurring in 43% of individuals between 65 and 74 years, and in as much as 88% of those above 85 years’ old.^5^

The association of memory complaints with depression, anxiety and personality traits is already well defined.^6,7^ Other factors such as female sex, low educational level^8^ and poor physical health^9,10^ have also been associated with memory complaints.

Several studies have attempted to evaluate how memory complaints relate to present or future cognitive impairment. However, controversy remains over the clinical meaning of such complaints.^1,3,8,10^ The majority of cross-sectional studies do not show association between cognitive complaints and cognitive performance as measured by psychometric tests.^11^ Some of these studies have shown the association between cognitive complaints and psychometric measures to be small or nonexistent ^6,10,12-14^ and that complaints were associated with depression,^6,13,15,16^ anxiety^6^ and personality traits.^6,7,17,18^ Carr and colleagues^19^ investigated a sample of individuals with very mild dementia of the Alzheimer type (DAT) containing no demented elderly subjects. They found no significant correlation between self-reported memory complaints and cognitive performance or further development of dementia. Informant-reported memory loss distinguished non-demented from demented individuals and predicted future diagnosis of DAT.

On the other hand, longitudinal studies have suggested that memory complaints could be associated to higher risk of cognitive decline.^20-26^ One such study observed an increased risk for the development of dementia among participants who presented lower cognitive performance, who were older and had more recent complaints.^21^ More recently, a study in elderly people with memory complaints but no dementia demonstrated that almost half of the evaluated participants displayed mild but measurable cognitive deficits.^27^ The authors stressed that memory complaints should not be underestimated and objective evaluation using psychometric tests allowed the identification of normal performance and possible cases of ‘mild cognitive impairment’ (MCI) in the elderly. At this juncture, the concept of MCI should be mentioned, a disease in which memory complaint is one of the core criteria for its identification.^28^ However, MCI remains a concept and not a specific diagnosis with the criteria proposed by the main authors in the field, and is not included in this format under any of the main diagnostic systems (i.e., DSM).

Nevertheless, there are other concepts similar to MCI, such as ‘age-associated memory impairment’^29^ and ‘ICD-10 Mild Cognitive Disorder’,^30^ which include memory complaints in their diagnostic criteria. Considering the rising interest in treating some of these conditions, it is very important to establish the relationship between cognitive complaints and current and future cognitive impairment.^31^

The “Mini-Mental State Examination” (MMSE) was designed to provide a mental state practical clinical assessment in geriatric inpatients^32,33^ and is one of the most widely used screening tests for this purpose. The MMSE is recommended as a screening tool for individuals with cognitive impairment^34,35^ and has been previously validated for use in Brazil.^36^

The goal of this study was to investigate the association between cognitive complaints and performance on a mental state screening test (the Mini mental State Examination – MMSE) in a sample of patients referred to the Neurogeriatric Clinic of a university hospital in Southern Brazil. It also analyzes the main reasons for referral and the rate of discharge from the Neurogeriatric Clinic.

## Methods

All patients referred to the Neurogeriatric and Dementia Outpatient Clinic from any other outpatient clinic within Hospital de Clínicas de Porto Alegre (HCPA), during 2005, 2006 and 2007 first semesters, were analyzed. The number of patients referred was 200.

This sample size was sufficient to detect memory complaints based on other studies that showed rates of 30%,^8^ a 2.0 relative risk of memory complaint for the development of Alzheimer’s disease and dementia,^26^ and a confidence interval of 95% (N=200).

The variables of interest were:

(a) source of and reason for referral (medical or surgical specialties that referred the patient for evaluation);
(b) whether or not they had previously been evaluated with the screening test (Mini Mental State Exam - MMSE) by their physicians before referral to our specialized clinic;
(c) cognitive complaint; and
(d) performance on the screening test (MMSE) at the NGA.

We also evaluated the demographic data of patients (age, sex, and educational level).

The MMSE^32,37-39^ was applied to all patients as a mental state screening test at the first NGA Clinic appointment. The adjusted-education cutoffs used were 24^37,38^ and 18.^39^ The initial evaluation also encompassed anamnesis, physical and neurological examination. An open questionnaire, applied during the medical history at the Neurogeriatric clinic, investigated cognitive complaints. All patients who fell into the category of “suspected dementia” were further evaluated using the standard protocol followed at the NGA Clinic for dementia. Other cases, *i.e.*, questionable dementia, were also followed at the Clinic until the resolution of this condition into dementia, normal or another category.

This study was approved by the Ethics and Research Committee of Hospital de Clínicas de Porto Alegre and all patients and their caregivers signed an informed consent form.

### Statistical analysis

Descriptive statistics (mean, SD, and frequency) were calculated for demographic data, and MMSE. Categorical data were analyzed with the Chi-square test, employing Yates correction to check association between them. The Mann-Whitney test was used to compare total MMSE score from subjects who presented cognitive complaints and from subjects who showed other complaints. The statistical analysis was performed by the *Statistical Package for the Social Sciences* - SPSS version 14.0.

## Results

From the 200 consecutive referrals to NGA Clinical, 130 subjects (65%) were female. Age was 67.0±14.1(mean±SD) years, education was 5.5±3.7(mean±SD) years, and total score on MMSE was 21.59±6.82(mean±SD) across the whole sample.

The main reason for referral to the NGA clinic was cognitive complaint 63% (N=126 from 200 subjects) (Table 1). Among subjects who showed cognitive complaints (N=126), isolated memory was the most frequent (N=111 out of 126, 88%), and memory associated to other cognitive complaints, such as orientation, language or executive functions, were responsible for the minority (N=15 out of 126, 12%) of complaints. Only 5% (N=10) of the referred patients had been previously evaluated by a cognitive screening test (MMSE or equivalent) (Table 1). During the first appointment at the NGA clinic, 68% (N=135) of the patients reported cognitive complaints, mainly involving memory (Table 2). During the first appointment at the NGA clinic, patients were assessed by the MMSE, on which 131 (65%) presented scores above the education-adjusted cut-off (Table 2).

**Table 1:** Reason for referrals in the sample.

Reason for referral	200 (100%)
Cognitive complaint	126 (63%)
Other neurological complaint	38 (19%)
Not registered/not found	36 (18%)
Patients submitted to a mental state screening test before referral (MMSE or equivalent)	10 (5%)

**Table 2:** Sample characteristics at the NGA clinic appointments (N=200).

	Frequency
Cognitive complaints at the first appointment at the NGA clinic	N=135 (68%)
Patients with MMSE scores above cut-off at the first appointment	N=131 (65%)
Source of referrals (medical specialties)	N=126 (63%)
Outcome: remained for follow-up	N=50 (25%)

Of the 135 patients who presented cognitive complaints during the first appointment, 52 (38%) presented MMSE scores below the education-adjusted cut-off (Table 3). No association between cognitive complaint and performance on the mental state screening test (MMSE) during the first evaluation at the NGA Clinic was observed (χ^2^=3.04, p=0.1).

**Table 3:** Association between cognitive complaint and performance on the mental state screening test (MMSE).

	Cognitive complaint (N=135)	Other neurological complaint (N=62)
Below cut-off (N=69)	52 (38%)	16 (26%)
Above cut-off (N=131)	83 (62%)	46 (74%)

Comparison between total MMSE score from subjects who presented cognitive complaints and from subjects who showed other complaints did not demonstrate significant difference (p=.43).

We observed a differential in rate of follow-up stay among patients in the cognitive complaints group, the other neurological complaint group, and also for the sample as a whole. Of the 200 patients referred to the NGA Clinic, 50 (25%) remained at the clinic for follow-up (Table 2). Of the 135 patients with cognitive complaint, N=46 (34%) stayed for follow-up, while of the 62 patients with other neurological complaint, only N=3 (5%) remained (Table 4). There was a significant association between cognitive complaint and staying at the NGA Clinic for follow-up (p<0.001). There was also a significant association between MMSE scores below cut-off and remaining at the NGA Clinic for follow-up (p<0.001).

**Table 4:** Analysis of cognitive complaint and MMSE scores below and above cutoff according to outcome on NGA clinic follow-up.

Complaint*	Follow-up at NGA clinic (N=49)	Discharged from NGA clinic (N=148)
Cognitive (N=135)	46 (34%)	89 (66%)
Other neurological (N=62)	3 (5%)	59 (95%)
MMSE**	(N=50)	(N=149)
Below cut-off (N=69 )	41 (82%)	28 (19%)
Above cut-off (N=130 )	9 (18%)	121 (81%)

* Chi-square=19.43; p<0.001 (Fisher exact);

** Chi-square=66.03; p<0.001 (Fisher exact).

Of the sixty three percent of the patients who were referred from the medical specialties, 27% were referred from surgical specialties, and 10% from Psychiatry. Among medical specialties, Neurology (15.8%) and Internal Medicine (14.6%) were those with the highest rates of referrals. There was no association between specialty and objective corroboration of cognitive impairment (p=0.9) or follow-up at the clinic (p=0.5). Gender did not present significant association to cognitive complaints (p=0.2). Age and education of the group of patients presenting cognitive complaints were not significantly different to the group presenting other neurological complaints (Table 5).

**Table 5:** Mean±SD of age and education of the cognitive complaint and other neurological complaint groups (Student's t test).

Variables	Cognitive complaint (N=135)	Other neurological complaint (N=62)	p value
Age	68.13±12.83	64.13±16.00	0.08
Education	5.5±3.7	5.5±3.5	0.88

## Discussion

The objective of this study was to analyze cognitive complaints in relation to the performance on a mental state screening test among elderly outpatients. We found no association between cognitive complaints and the Mini Mental State Exam in the sample of the present study. Our finding corroborates previous studies demonstrating that cognitive complaints were not associated to cognitive impairment, or to further development of dementia.^6,10,12-15^ In fact, complaints are often inconsistent with the actual levels of impairment and are frequently associated to psychiatric disorders.^6,7,17,20,40^ In addition, most dementia patients are not aware of their cognitive limitations.^22^ It is very important for physicians to understand that the diagnostic and predictive value of cognitive complaints for detection of cognitive impairment should not be overestimated. Other measures such as intra-individual and functional decline perceived and reported by informants can be better predictors of dementia^19^ and should always be investigated.

In contrast, other studies have found association between cognitive complaints and measurable cognitive deficit^20-26^ and have considered complaints a risk factor for development of dementia.^1,3,21,41-43^ One possible explanation for these contradictory findings is the way in which memory complaints are assessed, as they may be spontaneously reported or measured by questionnaires in epidemiological studies. Another methodological problem is whether or not these studies take into account variables such as depression, level of education and cognitive impairment, which might determine the association between complaints and performance.^8^

Another interesting finding of this study was that the majority of referrals took place solely because the patient had complained about their cognitive capacity to his/her physician and seldom as a result of mental state screening to check the complaint (only 5%). Many physicians do not routinely perform mental state examinations and may not be familiar with the parts of the mental state assessment that are most useful for early detection of dementia.^44-46^ Referring patients based only on cognitive complaints was possibly responsible for the low frequency of cases that were not discharged from the Neurogeriatric clinic. Cognitive screening tools like the MMSE, are brief, reliable and feasible instruments, and should be part of the routine examination for any older subject in any specialty.

Another study carried out in patients with different neurological conditions reinforced the importance of including cognitive evaluation as a routine part of the neurological evaluation.^47^ Furthermore, if complaints constitute the main reason for suspecting dementia and referring subjects to the Neurogeriatric clinic, many demented persons who do not actually perceive their deficits might go undiagnosed.

Age, sex and educational level were not associated to cognitive or to other neurologic complaints. One limitation of the present study was that association to other non-cognitive variables, such as anxiety, depression and cognition was not investigated, where this warrants further studies. We could also call into question the way cognitive complaints were verified because no specific instrument was used for validation. An open question during the medical history at the Neurogeriatric clinic investigated this issue, although all members of the team were trained for the standard history at the clinic.

There is some debate on sensitivity of the screening test for detection of cognitive impairment, mainly for subjects with higher educational level.^48^ In such cases, individuals can perceive change in their cognitive performance and complaints, but this decline is insufficient to produce measurable abnormalities in neuropsychological tests.^1^ By the same token, low education level must also be considered when general cognitive screening tests such as the MMSE are applied.^36,38,39,48,51-53^

Obviously, the current state of the art coupled with the serious repercussions of dementia diagnosis does not allow for a diagnostic process based on one single measure. However, it is very important to stress the development of “a geriatric consciousness”. Dementia is one of most frequent problems in older people^49,50^ and its incidence increases exponentially with age.^50^ Considering the epidemiology of dementia and worldwide ageing, we believe that mental state examination and the use of easy and feasible cognitive screening tools should be an integral part of the diagnostic instrument arsenal of any physician who deals with elderly patients.

## References

[1] Schofield PW, Jacobs D, Marder K, Sano M, Stern Y (1997). The validity of new memory complaints in the
elderly. Arch Neurol.

[2] Barker A, Jones R, Jennison C (1995). A prevalence study of age-associated memory
impairment. Br J Psychiatry.

[3] Tobiansky R, Blizard R, Livingston G, Mann A (1995). The Gospel Oak Study stage IV: the clinical relevance of
subjective memory impairment in older people. Psychol Med.

[4] Jonker C, Launer LJ, Hooijer C, Lindeboom J (1996). Memory complaints and memory impairment in older
individuals. J Am Geriatr Soc.

[5] Larrabee GJ, Crook TH III (1994). Estimated prevalence of age-associated memory impairment derived
from standardized tests of memory function. Int Psychogeriatr.

[6] Jorm AF, Christensen H, Henderson AS, Korten AE, Mackinnon AJ, Scott R (1994). Complaints of cognitive decline in the elderly:a comparison of
reports by subjects and informants in a community survey. Psychol Med.

[7] Kahn RL, Zarit SH, Hilbert NM, Niederehe G (1975). Memory complaints and impairment in the aged. Arch Gen Psychiatry.

[8] Jonker C, Geerlings MI, Schmand B (2000). Are memory complaints predictive for dementia? A review of
clinical and population based studies. Int J Geriatr Psychiatry.

[9] Stewart R, Russ C, Richards M, Brayne C, Lovestone S, Mann A (2001). Depression, APOE genotype and subjective memory impairment:a
cross-sectional study in an African-Caribbean population. Psychol Med.

[10] Comijs HC, Dik MG, Deeg DJ, Jonker C (2004). The course of cognitive decline in older persons: results from
the Longitudinal Aging Study Amsterdam. Dement Geriatr Cogn Disord.

[11] Riedel-Heller SG, Schork A, Matschinger H, Angermeyer MC (2000). Subjective memory loss-a sign of cognitive impairment in the
elderly? An overview of the status of research. Gerontol Geriatr.

[12] O'Hara MW, Hinrichs JV, Kohout FJ, Wallace RB, Lemke JH (1986). Memory complaint and memory performance in the depressed
elderly. Psychol Aging.

[13] O'Connor DW, Pollitt PA, Roth M, Brook PB, Reiss BB (1990). Memory complaints and impairment in normal, depressed and
demented elderly persons identified in a community survey. Arch Gen Psychiatry.

[14] Sunderland A, Watts K, Baddeley AD, Harris JE (1986). Subjective memory assessment and test performance in elderly
adults. J Gerontol.

[15] McGlone J, Gupta S, Humphrey D, Oppenheimer S, Mirsen T, Evans DR (1990). Screening for early dementia using memory complaints from
patients and relatives. Arch Neurol.

[16] Bolla KI, Lindgren KN, Bonaccorsy C, Bleecker ML (1991). Memory complaints in older adults: Fact or
fiction?. Arch Neurol.

[17] Poitrenaud J, Malbezin M, Guez D (1989). Self-rating and psychometric assessment of age-related changes in
memory among young-elderly managers. Dev Neuropsychol.

[18] Seidenberg M, Taylor MA, Haltiner A (1994). Personality and self-report of cognitive
functioning. Arch Clin Neuropsychol.

[19] Carr DB, Gray S, Baty J, Morris JC (2000). The value of informant versus individual's complaints of memory
impairment in early dementia. Neurology.

[20] Geerlings MI, Jonker C, Bouter LM, Ader HJ, Schmand B (1999). Association between memory complaints and incident Alzheimer's
disease in elderly people with normal baseline cognition. Am J Psychiatry.

[21] Treves TA, Verchovsky R, Klimovitzky S, Korczyn AD (2005). Incidence of dementia in patients with subjective memory
complaints. Int Psychogeriatr.

[22] Jorm AF, Christensen H, Korten AE, Jacomb PA, Henderson AS (2001). Memory complaints as a precursor of memory impairment in older
people:a longitudinal analysis over 7-8 years. Psychol Med.

[23] Wang L, van Belle G, Crane PK (2004). Subjective memory deterioration and future dementia in people
aged 65 and older. J Am Geriatr Soc.

[24] St John P, Montgomery P (2002). Are cognitively intact seniors with subjective memory loss more
likely to develop dementia?. Int J Geriatr Psychiatry.

[25] Dufouil C, Fuhrer R, Alperovitch A (2005). Subjective cognitive complaints and cognitive decline:
consequence or predictor? The epidemiology of vascular aging
study. J Am Geriatr Soc.

[26] Palmer K, Bäckman L, Winblad B, Fratiglioni L (2003). Detection of Alzheimer's disease and dementia in the preclinical
phase:population based cohort study. BMJ.

[27] Gallassi R, Bisulli A, Oppi F, Poda R, Di C (2008). Subjective cognitive complaints, neuropsychological performance,
affective and behavioral symptoms in non-demented patients. Int J Geriatr Psychiatry.

[28] Petersen RC, Smith GE, Waring SC, Ivnik RJ, Tangalos EG, Kokmen E (1999). Mild cognitive impairment: clinical characterization and
outcome. Arch Neurol.

[29] Crook T, Bahar H, Sudilovsky A (1987). Age-associated memory impairment: diagnostic criteria and
treatment strategies. Int J Neurol.

[30] World Health Organization (1992). The ICD-10 Classification of Mental and Behavioral Disorders. Clinical
Descriptions and Diagnostic Guidelines.

[31] Reid LM, MacLullich AMJ (2006). Subjective memory complaints and cognitive impairment in older
people. Dement Geriatr Cogn Disord.

[32] Folstein MF, Folstein SE, McHugh PR (1975). "Mini-Mental State":a practical method for grading the cognitive
state of patients for the clinician. J Psychiatr Res.

[33] Folstein M (1998). Mini-mental and son. Int J Geriatr Psychiatry.

[34] Nitrini R, Caramelli P, Bottino CM, Damasceno BP, Brucki SM, Anghinah R (2005). Diagnóstico de Doença de Alzheimer no Brasil.
Avaliação cognitiva e funcional. Recomendações
do Departamento Científico de Neurologia Cognitiva e do
Envelhecimento da Academia Brasileira de Neurologia. Arq Neuropsiquiatr.

[35] Petersen RC, Stevens JC, Ganguli M, Tangalos EG, Cummings JL, DeKosky ST (2001). Practice parameter: Early detection of dementia: Mild cognitive
impairment (an evidence-based review). Report of the Quality Standards
Subcommittee of the American Academy of Neurology. Neurology.

[36] Bertolucci PHF, Brucki SMD, Campacci SR, Juliano Y (1994). O mini-exame do estado mental em uma população
geral:impacto da escolaridade. Arq Neuropsiquiatr.

[37] Chaves ML, Izquierdo IA (1992). Differential Diagnosis between Dementia and Depression: a Study
of Efficiency Increment. Acta Neurol Scand.

[38] Almeida OP (1998). Mini exame do estado mental e o diagnóstico de
demência no Brasil. Arq Neuropsiquiatr.

[39] Caramelli P, Herrera JR E, Nitrini R (1999). O mini-exame do estado mental no diagnóstico de
demência em idosos analfabetos. Arq Neuropsiquiatr.

[40] Gagnon M, Dartiques JF, Mazaux JM (1994). Self-reported memory complaints and memory performance in elderly
French community residents results of the PAQUID research
program. Neuroepidemiology.

[41] Bassett SS, Folstein MF (1993). Memory complaint memory performance and psychiatric diagnosis a
community study. J Geriatr Psychiatry Neurol.

[42] O'Brien JT, Beats B, Hill K (1992). Do subjective memory complaints precede dementia? A three-year
follow-up of patients with supposed "benign senescent
forgetfulness". Int J Geriatr Psychiatry.

[43] Taylor JL, Miller TP, Tinklenberg JR (1992). Correlates of memory decline: a 4-year longitudinal study of
older adults with memory complaints. Psychol Aging.

[44] Knopman DS (1998). The initial recognition and diagnosis of Dementia. Am J Med.

[45] Callahan CM, Hendrie JC, Tierney WM (1995). Documentation and evaluation of cognitive impairment in elderly
primary care patients. Ann Intern Med.

[46] Ravdin LD, Mattis PJ, Lachs MS (2004). Assessment of cognition in primary care: neuropsychological
evaluation of the geriatric patient. Geriatrics.

[47] Vitiello APP, Ciríaco JGM, Takahashi DY, Nitrini R, Caramelli P (2007). Avaliação cognitiva breve de pacientes atendidos em
ambulatório de neurologia geral. Arq Neuropsiquiatr.

[48] Uhlmann RF, Larson EBL (1991). Effect of education on the mini-mental state examination as a
screening test for dementia. J Am Geriatr Assoc.

[49] Berr C, Wancata J, Ritchie K (2005). Prevalence of dementia in the elderly in Europe. Eur Neuropsychopharmacol.

[50] Rice DP, Fillit HM, Max W, Knopman DS, Lloyd JR, Duttagupta S (2001). Prevalence, costs, and treatment of Alzheimer's disease and
related dementia: a managed care perspective. Am J Manag Care.

[51] O'Connor DW, Pollitt PA, Treasure FP (1989). The influence of education, social class and sex on Mini-Mental
State scores. Psychol Med.

[52] Jorm AF, Scott R, Henderson AS (1988). Educational level differences on the Mini-Mental State: the role
of test bias. Psychol Med.

[53] Brucki SMD (1996). Mini-exame do estado mental: influência da escolaridade
sobre os escores total e sub-itens. Rev Neuroci.

